# Radiation‐Associated Breast Angiosarcoma Versus Recurrent Invasive Ductal Carcinoma After Partial Mastectomy: A Diagnostic Dilemma

**DOI:** 10.1002/ccr3.70967

**Published:** 2025-09-22

**Authors:** Hidetoshi Satomi, Ayumi Ryu, Sei Murayama, Yuki Morimoto, Chiaki Kubo, Shigenori Nagata, Satoshi Tanada, Keiichiro Honma

**Affiliations:** ^1^ Department of Diagnostic Pathology and Cytology Osaka International Cancer Institute Chuo‐ku Osaka Japan; ^2^ Department of Clinical Laboratory Osaka International Cancer Institute Chuo‐ku Osaka Japan

**Keywords:** angiosarcoma, cytomorphology, fine‐needle aspiration, histology

## Abstract

Postradiation angiosarcoma (PRAS) of the breast occurs after irradiation. It is categorized separately from primary angiosarcoma (PAS) in the 2019 World Health Organization classification of tumors. PRAS diagnosis is challenging owing to its occurrence in circumstances similar to breast cancer recurrence, thus complicating cytomorphological analysis. In Japan, breast‐conserving therapy is prevalent, with postoperative radiation therapy often employed to mitigate recurrence risk. Given that the number of PRAS cases is anticipated to increase, further research and understanding of this tumor are imperative. Although cytomorphological studies have provided some insights into PAS, similar comprehensive data for PRAS are lacking. In this case study, we report a case of a female in her 50s with a mass detected 8 years postradiotherapy for invasive ductal carcinoma (IDC). Fine needle aspiration cytology (FNAC) initially suggested IDC recurrence; however, histological and immunohistochemical analyses confirmed PRAS. This case highlights the challenge of distinguishing PRAS from IDC owing to overlapping cytomorphological features. Notably, the absence of benign components and distinctive endothelial wrapping observed on FNAC and imprinting cytology were crucial for accurate diagnosis. These findings highlight key cytomorphological features for PRAS differentiation: high‐grade tumor features with monotonous appearance and abundant stromal component, which are essential given its poor response to conventional treatments and increasing incidence owing to standard breast‐conserving therapies. Furthermore, recognizing PRAS as a differential diagnosis for neoplasms emerging postradiotherapy is crucial.

**Summary**

- Distinction between PRAS and IDC remains challenging due to overlapping features, resulting in low diagnostic accuracy.
- Identification of distinctive cytomorphological characteristics of PRAS, including endothelial wrapping and cytoplasmic vacuoles, is crucial for accurate diagnosis.

## 1 Introduction

Angiosarcoma, a rare tumor constituting 0.05% of all mammary malignancies, is classified into two distinct entities: postradiation angiosarcoma (PRAS) of the breast and primary angiosarcoma (PAS) in the 5th edition of the 2019 World Health Organization classification [1]. PAS is typically observed in individuals in their 20s. Conversely, PRAS manifests in individuals aged 60–70 years, with a latency period of 5–10 years postradiotherapy. However, despite existing reports on cytomorphological aspects of PAS, few reports exist regarding the cytomorphological analysis of PRAS of the breast, suggesting a gap in comprehensive knowledge of the disease [2].

Here, we report a case of angiosarcoma that developed after surgery and radiation therapy for invasive ductal carcinoma (IDC), focusing on the importance of cytology in diagnosing PRAS. This case may contribute to improved differential diagnosis and guide appropriate treatment strategies.

## 2 Case History/Examination

The patient was a woman in her 50s with no prior medical history. She underwent a partial mastectomy for IDC in the left C region. The postoperative diagnosis was pT1b, pN0, pStage I, and the surgical margin was 200 μm. The patient received 60 Gy/30 Fr of radiotherapy and five additional electron beam treatments. Eight years postradiation therapy, the patient observed a mass in the left anterior thoracic region, prompting fine‐needle aspiration cytology (FNAC).

## 3 Methods (Differential Diagnosis, Investigations, and Treatment)

**FIGURE 1:**
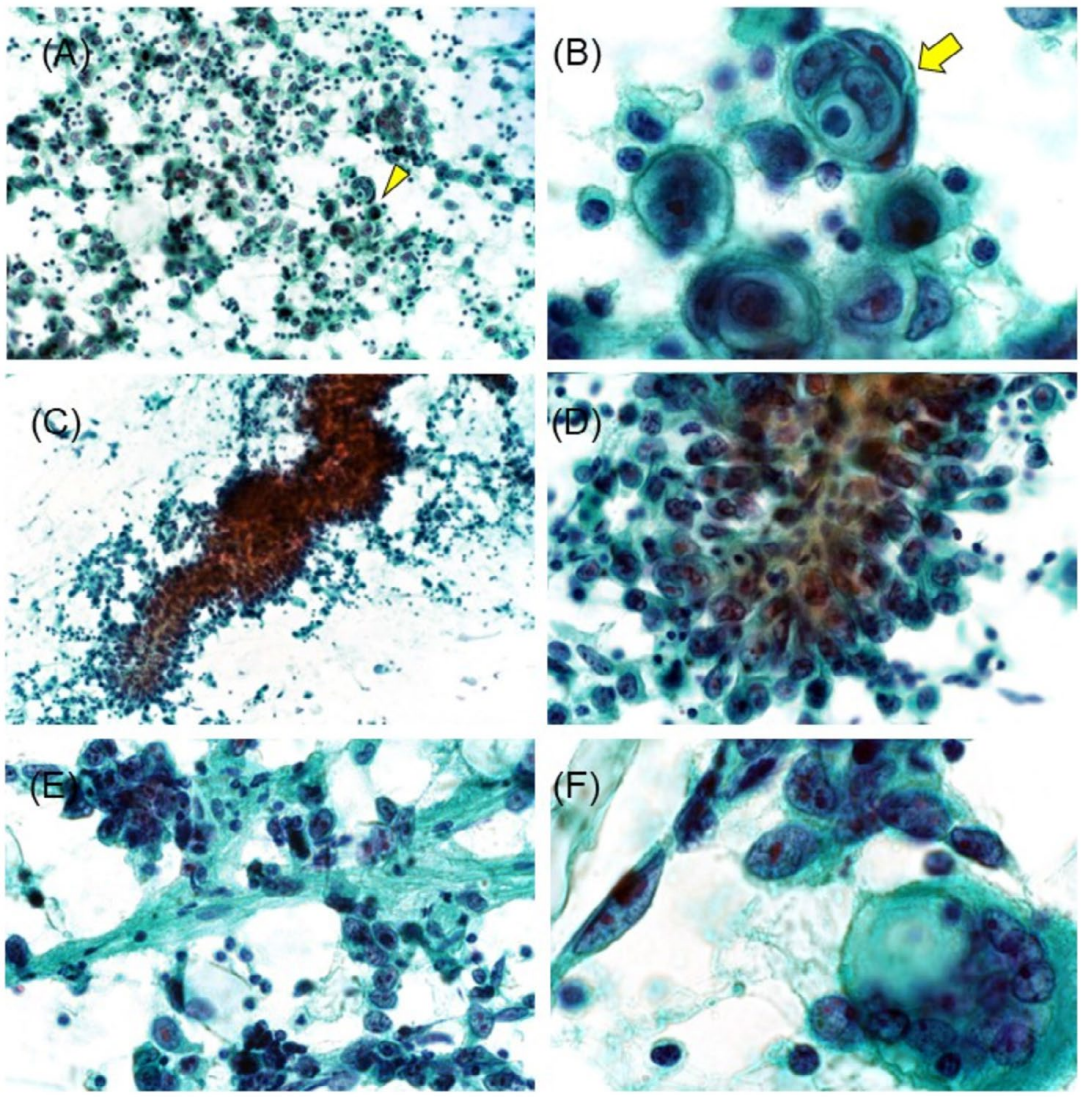
(A–D) Fine‐needle aspiration findings. (A) Atypical cells are solitary and scattered rather than clustered. (B) High magnification of atypical cells (arrowhead in A) reveals large cells with cytoplasmic vacuoles surrounded by flat cells, forming endothelial wrapping (arrow). (C, D) Tumor cells exhibit an intertwined distribution around the stromal axes. (E, F) Imprinting cytology from a surgical specimen. (E) Tumor cells are distributed around the blood vessels. (F) Endothelial‐like cells with nuclear atypia were observed. Cytoplasmic vacuoles are observed in the large, atypical cells. (A) ×200; (B, F) ×1000; (C) ×100; (D, E) ×400.

**FIGURE 2:**
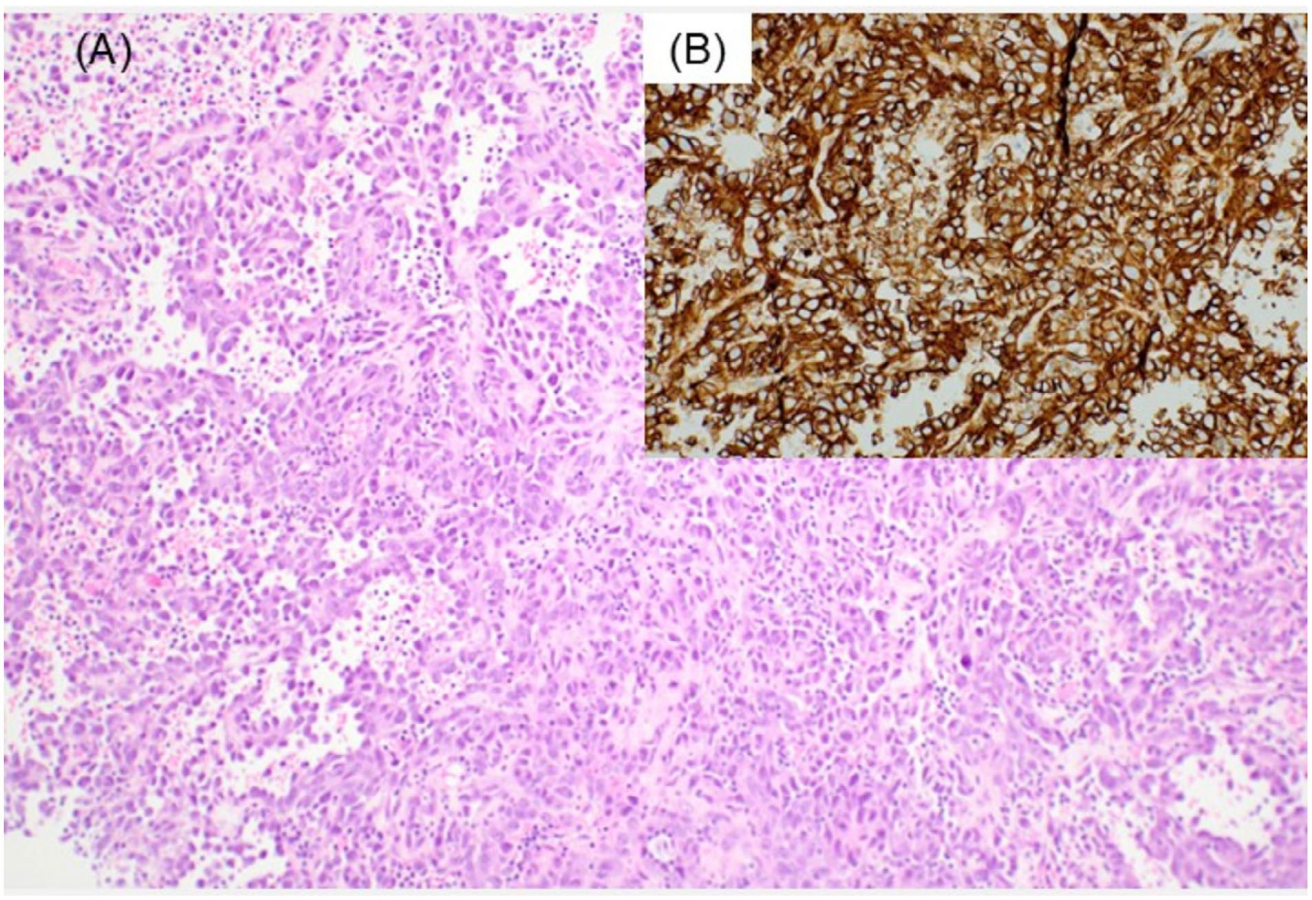
Total mastectomy findings. (A) Atypical cell proliferation forms solid or tubular structures with red blood cells in the lumen. (B) Immunohistochemical staining revealed CD31 positivity. (A) ×100; (B) ×200.

**FIGURE 3:**
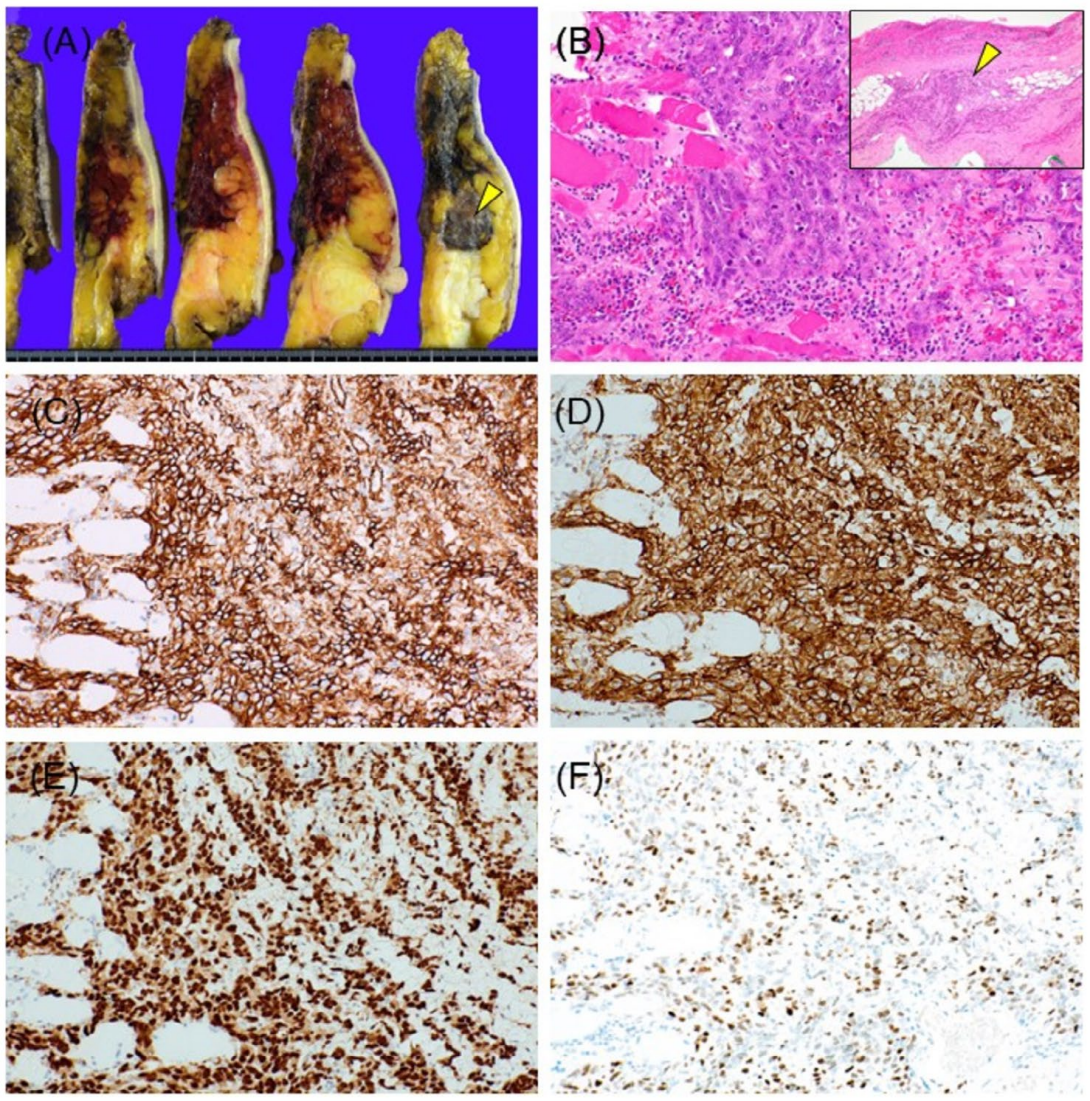
Total mastectomy findings. (A) Dark‐reddish tumor measuring approximately 23 × 21 mm, with map‐like extension. (B) Hematoxylin and eosin staining: Histologic findings of the area marked by the arrowhead in (A) show highly atypical cells with nuclear atypia, forming cordate or irregular solid structures. Slit‐like structures containing erythrocytes and prominent lymphocyte infiltration are observed, with tumor invasion into the pectoralis major muscle. Tumor cell infiltration is also seen in the intercostal muscles (arrowhead, inset). (C) Immunohistochemical staining reveals CD31 positivity in neoplastic cells. (D) D2‐40 positivity. (E) ERG positivity. (F) Predominant C‐Myc positivity. (B) ×100; (C–F) ×200.

FNAC revealed atypical cells clustered in an inflammatory background, predominantly composed of lymphocytes (Figure 1A). Rather than forming clusters, the cells displayed a disorganized interlacing arrangement around vascular structures or an axis of elongated stromal‐like cells, displaying numerous highly atypical features and conspicuous nucleoli (Figure 1C,D). Initially, these findings suggested IDC recurrence. However, histological analysis of the needle biopsy specimen revealed a proliferative pattern of atypical cells forming solid or tubular structures with red blood cells in the lumen. Immunohistochemical staining revealed CD31 and erythroblast transformation‐specific‐related gene positivity, confirming a diagnosis of angiosarcoma (Figure 2). Subsequently, a total mastectomy was performed. Macroscopically, a dark reddish hemorrhagic lesion with indistinct borders was observed in the left CD region, extending and oozing into the peri‐mammary tissue and skin (Figure 3A). Histologically, the lesion exhibited a solid component, with some papillary and tubular structures, consistent with the needle biopsy findings (Figure 3B). Immunohistochemically, the tumor cells were positive for vascular markers, including CD31, and c‐Myc was detected in most neoplastic cells (Figure 3C–F). Histologically, these findings were consistent with those of PRAS. No benign or transient sarcomatous components were observed.

Imprint cytology from the surgical specimen mirrored the preoperative FNAC, showing high‐grade atypical neoplastic cells with relatively monotonous morphology and no benign components such as hemangiomas. Some cells exhibited large cytoplasmic vacuoles (Figure 1F). A review of the original FNAC specimen revealed endothelial wrapping, with flat cells surrounding larger cells (Figure 1B). Retrospectively, the cytological findings were consistent with those of angiosarcoma.

## 4 Conclusions and Results (Outcome and Follow‐Up)

A total mastectomy was performed; the lateral skin excision margin was negative for tumor infiltration, but the lateral chest wall excision margin was positive; therefore, the intercostal muscles were additionally excised. One month later, a nodule appeared near the surgical scar. As the lesion was growing, postoperative chemotherapy was scheduled once a week with paclitaxel as the main drug. She is still continuing treatment.

## 5 Discussion

Most cytomorphological studies of breast angiosarcomas have focused primarily on PAS, with few reports on PRAS cytomorphology [3]. The key cytomorphological features for diagnosing breast IDC include nuclear atypia, loss of myoepithelial and glandular epithelial biphasic features, increased cell density, and decreased intercellular adhesion. Notably, these characteristics were evident in the current case, leading to an initial diagnosis of ID recurrence. However, upon histological diagnosis of angiosarcoma, a review of the FNAC findings revealed endothelial wrapping and large cytoplasmic vacuoles in the imprinting specimen, supporting a cytomorphological diagnosis of angiosarcoma.

The diagnostic accuracy of PRAS remains low, with Strobbe et al. [4] reporting a 22% diagnosis rate by FNAC. Although no comprehensive data exist on the correct diagnosis rate of PRAS of the breast, previous reports have highlighted the diagnostic challenges [5]. PRAS exhibits similar cytomorphological features to IDC, making the identification of distinctive characteristics crucial for avoiding misdiagnosis. Geller et al. [6] identified several characteristic features of angiosarcoma, including hemophagocytosis (54%), cytoplasmic lumina/vacuoles (69%) containing red blood cells (54%) or neutrophils (31%), and endothelial wrapping (69%). They reported that 88% of angiosarcomas exhibit one of these features. However, 12% lack these characteristics, indicating a need for further investigation [6]. In our case, in addition to endothelial wrapping and cytoplasmic lumina/vacuoles, the cytological findings revealed a distinctive pattern of intertwining growth around the fibrous stroma and blood vessels. This finding, which is not typical of IDC, may be unique to angiosarcoma and reflect its histologic features. Notably, the mammary gland, rich in collagen fibers, may have influenced the cellular morphology of angiosarcoma, mirroring the original breast lesion. Furthermore, the absence of a benign component in both the FNAC and imprinting cytology is atypical for PAS, which generally contains a transitional benign component. Therefore, the diffuse distribution of malignant cells may represent a characteristic feature of PRAS.

In mammary angiosarcomas, positive immunohistochemical staining of c‐Myc is valuable for distinguishing PAS from PRAS [7]. C‐Myc positivity is rare in PAS but relatively common in PRAS, suggesting that *MYC* mutations contribute to PRAS development. Furthermore, PAS often features a mingled hemangioma component or migrates from existing hemangiomas, whereas PRAS may develop de novo [8, 9, 10]. This difference may explain the more diffuse and malignant cytomorphological appearance of PRAS. Integrating these findings with existing cytomorphological data on angiosarcoma could facilitate an accurate diagnosis of PRAS in the mammary gland and ensure it is considered in the differential diagnosis.

Accurately distinguishing between angiosarcoma (PAS and PRAS) and IDC is essential given the differing treatment approaches. From the perspective of the surgery, unlike IDCs, which often form localized lesions, angiosarcomas, especially PRAS, are difficult to determine in terms of extent, making complete resection challenging [1]. From a pharmacotherapy perspective, IDC primarily requires an endocrine therapy of choice, whereas PRAS does not, although there is an ongoing debate regarding the efficacy of adjuvant chemotherapy in treating angiosarcoma [11]. Thus, accurate differentiation is essential for appropriate treatment selection. Therefore, differentiating complex cases from IDC is critical. Considering that breast‐conserving treatment and postoperative radiotherapy are now becoming standard treatment approaches, the incidence of PRAS is anticipated to increase [12]. Therefore, recognizing PRAS as a potential differential diagnosis for breast masses is critical. Specifically, if cytological specimens from the breast postradiation exhibit a monotonous, high‐grade, diffuse tumor with abundant stromal components, PRAS rather than IDC recurrence should be considered.

## Author Contributions

**Hidetoshi Satomi:** conceptualization, data curation, writing – original draft. **Ayumi Ryu:** conceptualization, data curation, writing – original draft. **Sei Murayama:** data curation. **Yuki Morimoto:** data curation. **Chiaki Kubo:** data curation, writing – review and editing. **Shigenori Nagata:** supervision. **Satoshi Tanada:** supervision. **Keiichiro Honma:** supervision.

## Ethics Statement

This case report was approved by the Institutional Review Board at Osaka International Cancer Institute (no. 24064).

## Consent

Written informed consent was obtained from the patient, and the report was approved by the appropriate ethics review board.

## Conflicts of Interest

The authors declare no conflicts of interest.

## Data Availability

The data that support the findings of this study are available from the corresponding author upon reasonable request.
